# Pulmonary Actinomycosis Induced by Total Gastrectomy and Heavy Alcohol Drinking: A Case Report

**DOI:** 10.7759/cureus.76398

**Published:** 2024-12-26

**Authors:** Sana Ohnuma, Keita Takizawa, Kana Ozasa, Jumi Nakata, Noboru Noma

**Affiliations:** 1 Department of Oral Medicine, Nihon University School of Dentistry, Tokyo, JPN; 2 Division of Internal Medicine, Towa Hospital, Tokyo, JPN

**Keywords:** chronic bronchitis, heavy alcohol consumption, oral hygiene, pulmonary actinomycosis, total gastrectomy

## Abstract

Combined risk factors such as total gastrectomy, heavy alcohol consumption, smoking, and poor oral hygiene may contribute to the development of pulmonary actinomycosis. Here, we present a rare case of pulmonary actinomycosis triggered by total gastrectomy and heavy alcohol consumption. The patient presented with hemoptysis and a suspected lung mass. With a history of total gastrectomy and heavy alcohol consumption, the patient underwent imaging studies that initially suggested lung cancer. While imaging studies initially suggested lung cancer, multiple bronchoscopic examinations ultimately led to a diagnosis of pulmonary actinomycosis, following an initial biopsy that revealed chronic bronchitis. The patient was treated with oral amoxicillin (1,500 mg/day for six months) alongside comprehensive dental care, including periodontal therapy and dental restorations. This approach resulted in significant clinical improvement, with no recurrence of hemoptysis and favorable findings on chest X-ray. This case highlights the intricate interplay of risk factors in the development of pulmonary actinomycosis. It underscores the effectiveness of a multidisciplinary treatment strategy combining antibiotic therapy and dental care, which improved the patient’s condition and avoided unnecessary interventions such as lung resection.

## Introduction

Actinomycosis is a systemic infection caused by the gram-positive rod bacteria of the genus Actinomyces, characterized clinically by chronic abscess formation, fistulas, and inflammatory lesions with firm fibrosis [1]. *Actinomyces israelii *is a major pathogen responsible for this condition, which is typically found as a normal flora in the oral cavity, gastrointestinal tract, and urogenital system [2]. Infection occurs when there is a breakdown in local defence mechanisms due to periodontal disease, trauma, or surgical interventions.

The onset of actinomycosis has been reported to be associated with risk factors such as poor oral hygiene and immune system dysfunction [3]. Additionally, heavy alcohol consumption and total gastrectomy are recognized as significant risk factors [4-6]. Heavy drinking not only impairs immune function but also adversely affects swallowing ability, increasing the risk of aspiration. This can elevate the likelihood of oral Actinomyces reaching the lungs and causing pulmonary actinomycosis [7]. Furthermore, total gastrectomy has been reported to lead to reduced lower esophageal sphincter (LES) function, making gastroesophageal reflux more likely and thereby increasing the risk of gastroesophageal reflux disease (GERD) [5,6]. This case report describes a patient with pulmonary actinomycosis triggered by significant risk factors, including total gastrectomy and heavy alcohol consumption. We present this case with a review of the literature to highlight the clinical features, diagnostic, and therapeutic importance of pulmonary actinomycosis.

## Case presentation

A 61-year-old male presented with hemoptysis, which developed one month after an abnormal shadow was detected in the right middle lung lobe on chest X-ray, leading him to visit the internal medicine outpatient clinic at a related hospital. A chest CT scan showed pleural traction in the right upper lung lobe near the hilum, strongly suggesting lung cancer, prompting further investigation with bronchoscopy for blood tests and tissue biopsy. His past medical history included total gastrectomy, cholecystectomy, splenectomy, D2 lymphadenectomy, and Roux-en-Y reconstruction for gastric cancer. Despite a history of heavy smoking (20 cigarettes/day since age 16) and daily alcohol consumption, the patient was not taking any regular medications, and his family history was unremarkable.

The oral examination revealed a large amount of plaque accumulation, caries, and gingival redness and swelling. The patient also had multiple missing teeth, resulting in unstable occlusion. There was no fever, and the patient reported no respiratory distress.In the initial blood test, only the white blood cell count (WBC) was elevated at 8,700/μL, above the normal range, while hemoglobin (Hb) was 12.1 g/dL and lactate dehydrogenase (LD) was 109 IU/L, both below the normal range. Red blood cell count (RBC) was 3.96 million/μL, platelet count (Plt) was 298,000/μL, total protein (TP) was 6.9 g/dL, blood urea nitrogen (BUN) was 19.8 mg/dL, creatinine (Cr) was 0.58 mg/dL, C-reactive protein (CRP) was 0.1 mg/dL, aspartate aminotransferase (AST) was 15 IU/L, alanine aminotransferase (ALT) was 11 IU/L, and creatine kinase (CK) was 114 IU/L, all within normal ranges. Tumor markers carcinoembryonic antigen (CEA) was 1.7 ng/mL and carbohydrate antigen 19-9 (CA19-9) was 27 U/mL, both also within normal ranges (Table 1).

**Table 1:** The results of blood tests at the first visit RBC: Red blood cell count, WBC: white blood cell count, PLT: platelet, HBG: hemoglobin, TP: total protein, BUN: blood urea nitrogen, Cr: creatinine, AST: aspartate aminotransferase, ALT: aspartate aminotransferase, LD: lactate dehydrogenase, CK: creatine kinase, CRP: C-reactive protein, CEA: carcinoembryonic antigen, CA19-9:  carbohydrate antigen 19-9.

Laboratory parameters with units	Patient values	Reference range
RBC (104/μL)	396	353-466
WBC (103/μL)	8.7	3.0-7.8
PLT (104/μL)	29.8	13.8-30.9
HGB (g/dL)	12.1	10.6-14.4
TP (g/dL)	6.9	6.7-8.3
BUN (mg/dL)	19.8	7-24
Cr (mg/dL)	0.58	0.61-1.04
AST (IU/L)	15	≦ 30
ALT (IU/L)	11	≦ 30
LD (IU/L)	109	119-229
CK (IU/L)	114	45-163
CRP (mg/dL)	0.1	≦ 0.3
CEA (ng/mL)	1.7	≦ 5.0
CA19-9 (U/mL)	27	≦ 37

**Figure 1:**
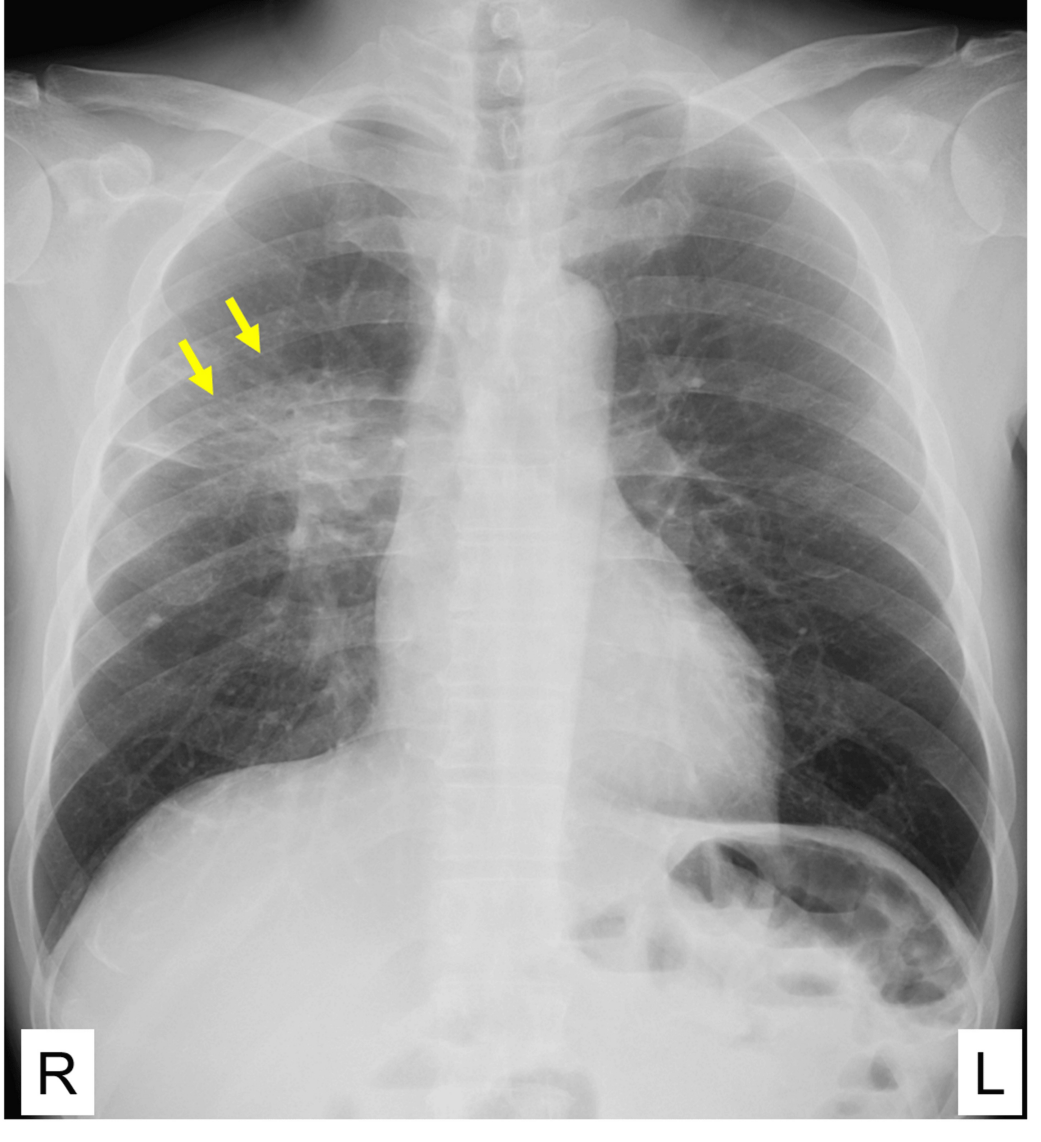
Chest X-ray of the chest at the first visit An abnormal nodular shadow was observed in the right middle lung field, along with prominence of the right hilar region, and lung consolidation in the lower part of the right hilus. R: right L: left.

The chest X-ray showed an abnormal nodular shadow in the right middle lung field (Figure 1).

**Figure 2:**
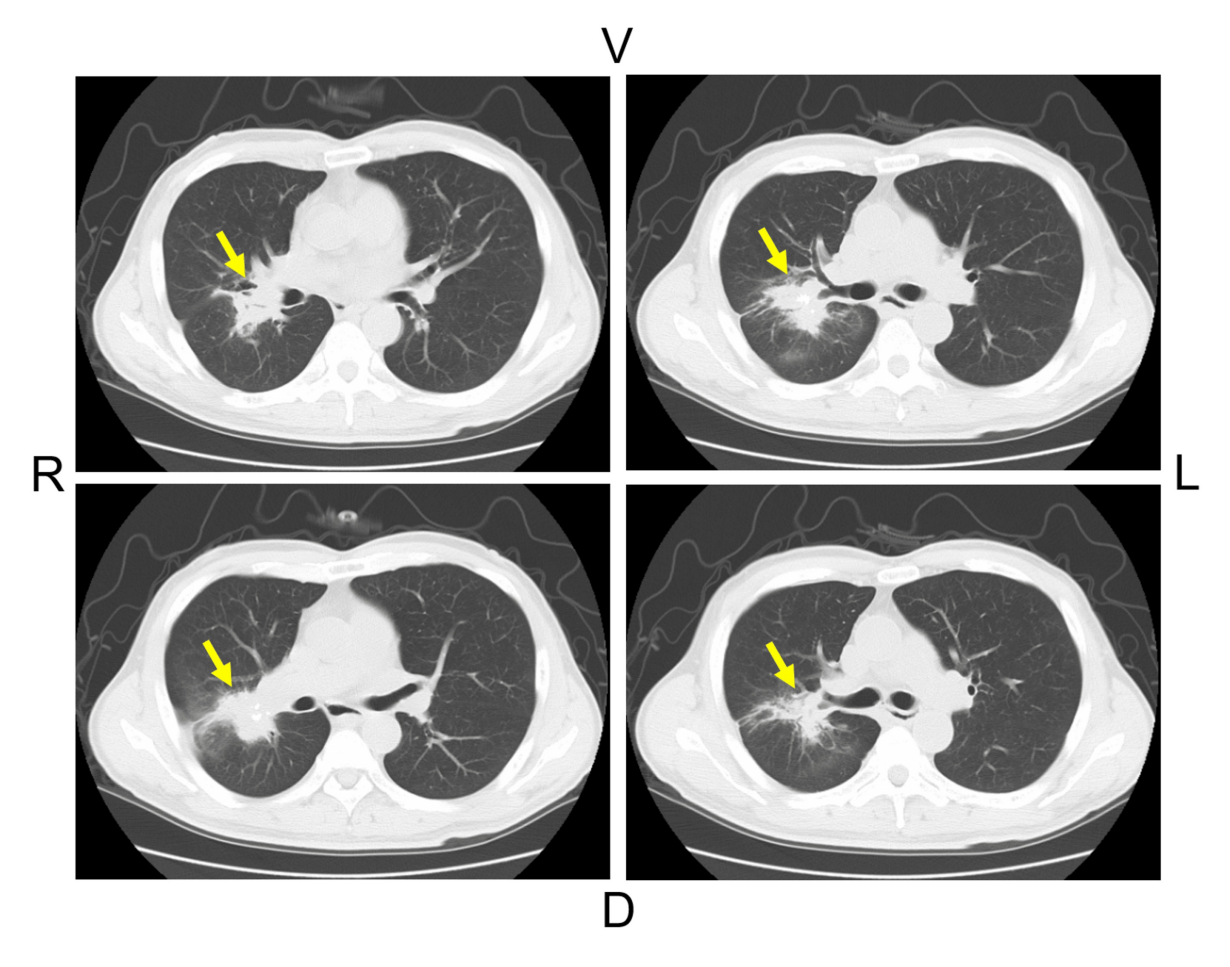
Contrast-enhanced computed tomography (CT) of the chest A nodular shadow with pleural traction and pleural retraction was observed in the right hilar region. R: right, L: left, V: ventral, D: dorsal.

The chest CT revealed pleural traction in the right lung hilum (Figure 2).　

**Figure 3:**
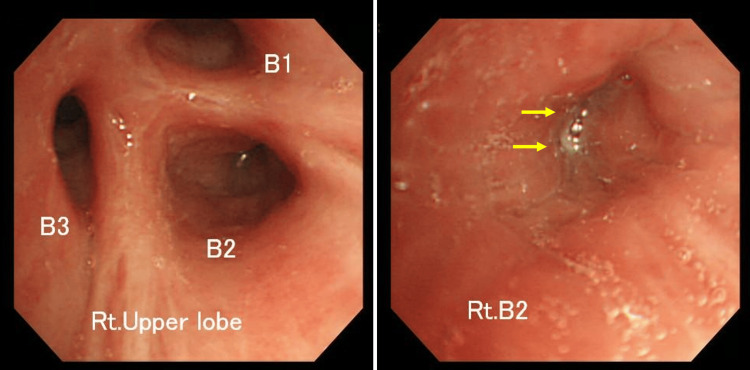
Bronchoscopy findings. It revealed swelling in the right upper lobe bronchus, with a reduction in the swelling noted but accompanied by purulent discharge. Rt. Upper lobe: right upper lobe, B1: apical segmental bronchus, B2: posterior segmental bronchus, B3: anterior segmental bronchus, Rt. B2: right posterior segmental bronchus.

Bronchoscopy revealed swelling in the right upper lobe bronchus, with a reduction in the swelling noted but accompanied by purulent discharge (Figure 3).

**Figure 4:**
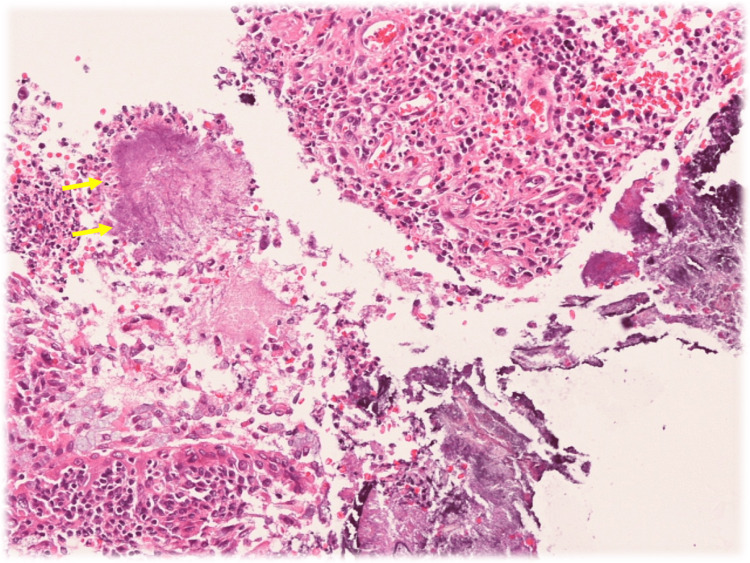
The fifth biopsy findings Biopsy samples from the first to the fourth procedures showed findings of chronic inflammation, including abscess formation and granulation tissue. The fifth biopsy revealed no cancer cells, but numerous clusters of radial filamentous structures, characteristic of Pulmonary Actinomycosis, were observed around the lesion.

The first four biopsies showed chronic bronchitis but did not reveal any findings suggestive of cancer; however, the fifth biopsy revealed numerous clusters of radial and filamentous structures, leading to a final diagnosis of pulmonary actinomycosis (Figure 4).

**Figure 5:**
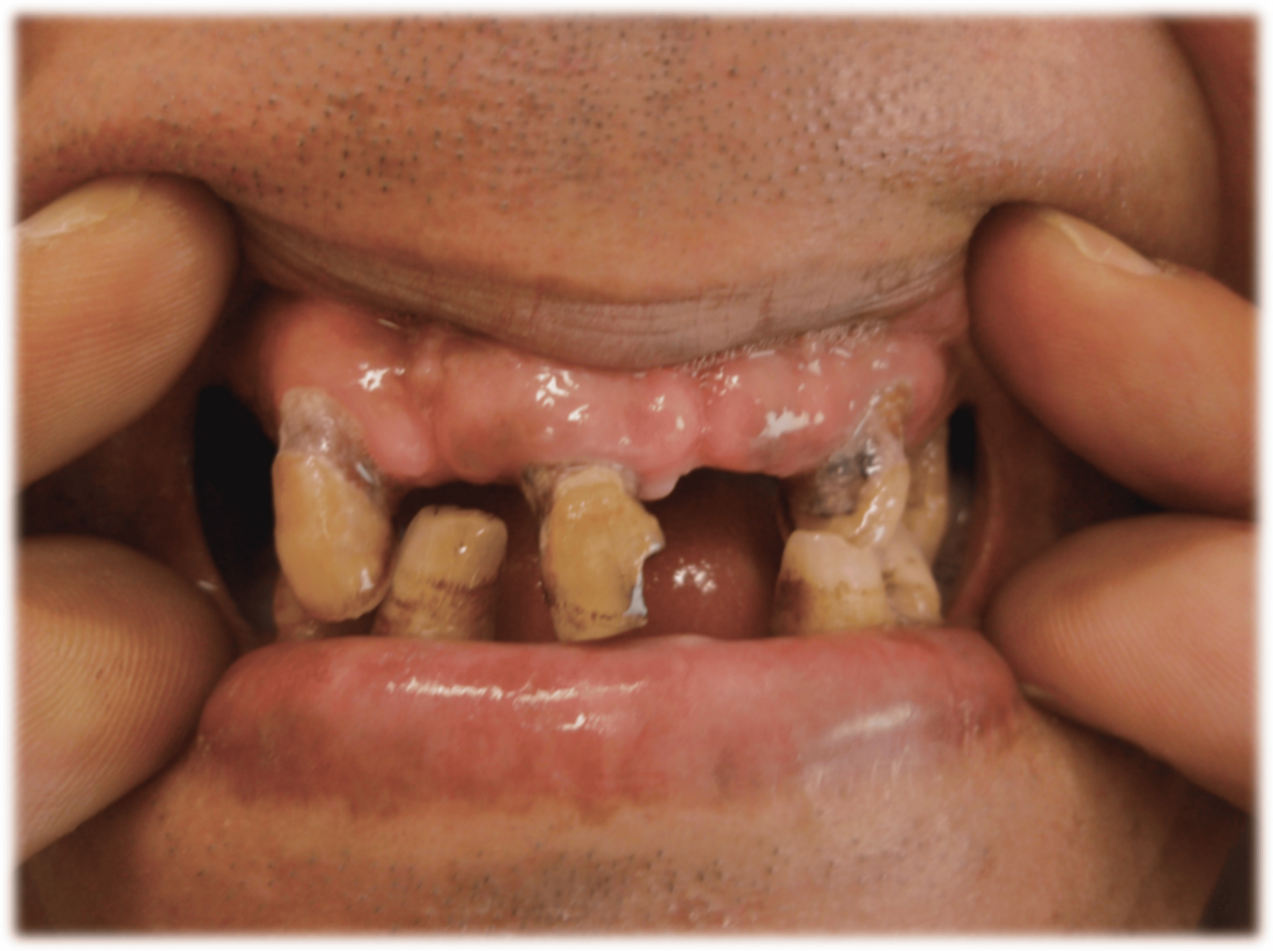
Oral Findings Large amount of plaque accumulation, caries, and gingivitis and resulting in unstable occlusion multiple caused by a lot of missing teeth. The Gingival Index (GI) was evaluated for seven teeth, each scoring 2 points, resulting in a total score of 16. The O'Leary Plaque Control Record (PCR) showed plaque on all 36 tooth surfaces, resulting in a PCR score of 100%.

A large amount of plaque accumulation, caries, and gingival redness and swelling. The patient had multiple missing teeth, resulting in partially edentulous upper and lower arches that caused unstable occlusion with crossbite and malocclusion.(Figure 5).

**Figure 6:**
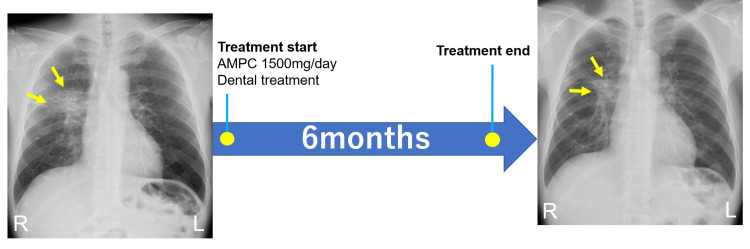
Treatment time course. The final diagnosis of pulmonary actinomycosis was followed by oral administration of AMPC 1,500 mg/day (two tablets, three  times daily), concurrent dental treatments. After six months, there was no hemoptysis, and although scar formation was observed on the chest X-ray, improvement in the lung consolidation and significant reduction of the right lung lesion were noted, indicating a favorable clinical outcome. R: right L: left, AMPC: amoxicillin.

Due to a strong suspicion of lung cancer based on chest X-ray and CT findings, bronchoscopy was performed for biopsy, revealing bronchial swelling and purulent discharge, but only chronic bronchitis was identified in the pathological findings. Subsequently, a total of five bronchoscopy procedures were conducted. By the fifth procedure, the bronchial swelling had reduced, and the pathological findings showed numerous clusters of radial and filamentous structures, leading to a diagnosis of pulmonary actinomycosis. The treatment involved oral administration of amoxicillin (AMPC) 1,500 mg/day (two tablets, three times daily) for six months. During this period, concurrent dental treatments were also performed, including periodontal therapy, extraction of remaining roots, endodontic therapy, and prosthetic treatment. After six months, there was no hemoptysis, and although scar formation was observed on the chest X-ray, improvements in abnormal findings such as lung consolidation and a significant reduction in the right lung lesion were noted, indicating a favorable clinical outcome (Figure 6).　

## Discussion

Actinomycosis, particularly pulmonary actinomycosis, presents a diagnostic challenge due to its similarity with other pulmonary infections and malignancies [8]. Pulmonary actinomycosis often manifests with non-specific symptoms such as persistent cough, fever, and weight loss, which are also common to other diseases, complicating the diagnostic process. While imaging studies and culture tests are critical for diagnosis, imaging frequently poses issues in differentiating pulmonary actinomycosis from malignant tumors. Therefore, careful consideration and a comprehensive diagnostic approach are essential for accurate identification of pulmonary actinomycosis.

CT scans and chest X-rays often reveal pulmonary abscesses or fibrotic lesions, which can be similar in appearance to malignancies. In this case, CT imaging initially suggested cancer; however, pulmonary actinomycosis was ultimately diagnosed after the fifth bronchoscopy biopsy. This example underscores that, due to the similarity in imaging findings between pulmonary actinomycosis and lung cancer, clinical symptoms and biopsy results play a crucial role in the diagnostic process.

According to our data, a total of 15 cases of difficult diagnosis between pulmonary actinomycosis and lung cancer have been reported over the past five years, including our own cases (Table 2) [1, 9-20].

**Table 2:** Report of Pulmonary actinomycosis similar to lung cancer in the past 5 years. This report examines the clinical findings from 15 cases, categorizing the associated symptoms and their examinations. M: male, F: female, L: left, R: right, B: bilateral, CT: computed tomography, CECT: contrast enhancement computed tomography, 18F-FDG PET/CT: 18F-fluorodeoxyglucose positron emission tomography / computed tomography, CTA: computed tomography angiography, AFB: acid fast bacteria, 68Ga-FAPI PET/CT: 68-galium fibroblast activation protein inhibitor positron emission tomography/computed tomography, AMPC: amoxicillin.

Author	Patient Age/Gender	Side	Symptoms	Examination	Diagnosis	Treatment
Tanaka.S et al. [9]	60/M	L	Chest pain during inspiration	Chest X-ray, Chest CT, Chest CECT, Biochemical studies, 18F-FDG PET/CT, CT-guided biopsy, Bronchoscopy	Pulmonary Actinomycosis	Tazobactam/piperacillin intravenouslyfot the 2 weeks, and after oral piperacillin
Asif AA et al. [10]	75/F	R	Fever and dry cough	Chest X-ray, Chest CT, Outpatient bronchoscopy with biopsy, CT-guided biopsy, Laboratory evaluation, Chest CTA	Pulmonary coccidioidomycosis with actinomycosis	Antimicrobial therapy with liposomal amphotericin and oral AMPC, dose of oral fluconazole
Cliffe A et al. [11]	72/F	B	Feeling generally unwell	CT, 18F-FDG PET/CT, Biopsy, Pulmonaly CTA	Actinomycosis and concurrent lung adenocarcinoma in situ	6 months of AMPC
Miyazaki S et al. [1]	64/M	L	Painful solid lump in the chest wall	Chest CT,18F-FDG PET/CT, Bronchoscopy, CT-guided transthoracic biopsy, Surgical resection	Pulmonary actinomycosis	8 week course of antibiotic therapy
Thapa K et al. [12]	70/M	R	Thoracoabdominal mass with shortness of breath, cough and 130 pound unintentional weight loss in the previous year	Chest CT, CT-guided biopsy, 18F-FDG PET/CT	Thoracoabdominal actinomycosis	6 weeks of IV penicillin, oral AMPC, oral doxycycline 100 mg two times per day, which he continued for 2 years
Aydin Y et al. [13]	54/F	L	Cough, fever, sputum, chest pain, and hemoptysis	18F-FDG PET/CT, Bronchoscopy, Tru-cut biopsy, Histopathological evaluation	Pulmonary actinomycosis	No data
Afsin E et al. [14]	65/M	R	Chronic cough	Chest X-ray, Blood test, Chest CT, Sputum AFB search, Fungal direct examination, Bronchoscopy, Biopsy	Pulmonary actinomycosis	AMPC-clavulanic acid was administered 3 g/day for one month and then continued as 2 g/day
Ferreira M et al. [15]	68/M	R	Anorexia and weight loss	Chest CT, Blood test, Transbronchial biopsy, 18F-FDG PET/CT, Transthoracic needle biopsy	Pulmonary actinomycosis	Intravenous amoxicillin and clavulanate for two weeks, followed by six months of oral treatment
Rouis H et al. [16]	70/M	L	Purulent productive cough and weight loss	Chest X-ray, Blood test, Sputum AFB search, Cytobacteriological sputum analysis, Chest CT, Bronchoscopy, Bronchial biopsy, Histological examination of the surgical resection	Pulmonary actinomycosis	AMPC-clavulanic acid for five weeks
Rouis H et al. [16]						
	39/M	R	Chest pain, hemoptysis, asthenia, and appetite loss			18 million units of penicillin G per day for 3 weeks intravenously followed by oral therapy with AMPC 1 g 3 times daily for 9 months
Cuzzani G et al. [17]	70/M	R	Hemoptysis	Thoracic high-resolution CT, 18F-FDG PET/CT, 68Ga-FAPI PET/CT, Bronchoscopy, Microbiology and biochemical blood test, Histopathologic, Immunohistochemistry	Pulmonary Actinomyces	no data
Bhat A et al. [18]	55/F	R	Hemoptysis and cough for three weeks	Respiratory examination, Cardiovascular and central nervous system examination, Segmental resection, Gross pathology, Microscopy	Bronchiectatic actinomycosis with osseous metaplasia	Prolonged antibiotics
Zheng M et al. [19]	70/M	R	Recurrent cough and sputum	Chest CT, Chest CECT, Ultrasound-guided lung biopsy, Microbiological examination	Pulmonary actinomycosis	Penicillin G at a dose of 720 units/day for 2 months intravenous, penicillin G on an outpatient basis for 6 months
Wu S et al. [20]	67/F	R	1-month history of coughing and expectoration	Chest CT, Chest CECT, PET-CT, Histopathological examination	Pulmonary actinomycosis	Right upper lobe resection and bronchoplasty, Oral AMPC for 2 months
Our case (Ohnuma et al.)	61/M	R	Hemoptysis	Blood test, Chest X-ray, Chest CT, Bronchoscopy, Biopsy	Pulmonary actinomycosis	Oral administration of AMPC 1,500 mg/day (2 tablets, 3 times daily) for six months, Dental treatment

The average age of the patients was 64.0 years, with 10 cases involving males and five involving females. The site of onset was the right side in 10 cases, the left side in four cases, and bilateral in one case. This predominance on the right side is attributed to the branching angle of the right bronchus being closer to vertical compared to the left, as well as the larger diameter of the right bronchus.

The most common symptom was cough, observed in 8 cases, followed by hemoptysis (five cases), chest pain (four cases), sputum (three cases), weight loss (three cases), and fever (two cases). Radiological examinations frequently included chest X-ray, CT, contrast-enhanced CT (CECT), and FDG-PET scans. Recent reports have described cases of pulmonary actinomycosis mimicking lung cancer on FDG-PET. These studies concluded that there is no significant difference in PET/CT findings between actinomycosis and lung cancer, limiting the diagnostic value of FDG-PET in differentiating the two conditions.

In some cases, lung resection was performed due to false-negative results in culture tests, radiological examinations, and bronchoscopic biopsies. Clinically, distinguishing between pulmonary actinomycosis and lung cancer remains a significant diagnostic challenge.

In the treatment of pulmonary actinomycosis, some clinicians recommend lung resection in the presence of symptoms such as sputum, hemoptysis, fever, and weight loss. However, while lung resection may be employed as a diagnostic and therapeutic intervention, it can sometimes lead to overtreatment [1]. In this case, the patient was treated with oral amoxicillin (AMPC) 250 mg for six months, in addition to concurrent dental treatment, resulting in significant symptom improvement. This suggests that lung resection could have been avoided with appropriate antibiotic therapy and dental care.

The causes of pulmonary actinomycosis include chronic oral infections, history of trauma or surgery, systemic diseases, smoking, prolonged use of antibiotics, periodontal disease, dental caries, and immunosuppressive conditions [3]. This case presents a patient with a history of total gastrectomy due to gastric cancer, and a lifestyle characterized by smoking (20 cigarettes/day), heavy alcohol consumption (daily), and extremely poor oral hygiene who developed pulmonary actinomycosis. The development of pulmonary actinomycosis in this case might be influenced by a combination of multiple risk factors.

Firstly, total gastrectomy leads to decreased function of the lower esophageal sphincter (LES) and facilitates the reflux of gastric acid. This reflux increases the likelihood of developing gastroesophageal reflux disease (GERD), which causes refluxed material to ascend from the esophagus to the oral cavity. When this refluxed material is aspirated, it can carry the actinomycetes that are normally present in the oral cavity into the airway, thereby increasing the risk of pulmonary involvement. Literature reports that GERD following total gastrectomy can promote the entry of oral flora into the airway, thereby elevating the risk of pulmonary infections [5,6].

Secondly, the patient's smoking habit and heavy alcohol consumption contribute to immune system suppression and impaired swallowing function. Smoking is known to have a suppressive effect on the immune system and compromise the defensive mechanisms of the respiratory system. Heavy alcohol consumption not only induces immunosuppression but also impairs swallowing function, thereby increasing the risk of aspiration. This, in turn, facilitates the easier transfer of actinomycetes from the oral cavity to the lungs [4,5,6].

Furthermore, poor oral hygiene promotes the proliferation of actinomycetes, thereby increasing the risk of their invasion into the lungs. Poor oral hygiene leads to excessive growth of oral flora, including actinomycetes, which increases the likelihood of aspiration and subsequent pulmonary invasion [6].

The interaction of these factors is believed to have contributed to the development of pulmonary actinomycosis in this case. The combination of GERD resulting from total gastrectomy, smoking, heavy alcohol consumption, and poor oral hygiene significantly increased the risk of actinomycete invasion into the lungs. It is therefore considered that these combined factors led to the onset of pulmonary actinomycosis.

## Conclusions

This case underscores the complex interplay of multiple risk factors in the development of pulmonary actinomycosis. The patient's history of total gastrectomy, smoking, heavy alcohol consumption, and poor oral hygiene collectively contributed to an increased risk of actinomycete invasion into the lungs. Despite initial imaging suggesting malignancy, the diagnosis of pulmonary actinomycosis was confirmed through biopsy after multiple bronchoscopies. Effective treatment with oral amoxicillin and concurrent dental care led to significant improvement, highlighting the importance of a comprehensive approach to diagnosis and management, potentially avoiding overtreatment such as lung resection.

## References

[1] Miyazaki S, Fujito T, Kondo Y (2022). Pulmonary actinomycosis mimicking lung cancer on (18)F-fluorodeoxyglucose positron emission tomography: a case report. J Med Case Rep.

[2] Wong VK, Turmezei TD, Weston VC (2011). Actinomycosis. BMJ.

[3] Valour F, Sénéchal A, Dupieux C (2014). Actinomycosis: etiology, clinical features, diagnosis, treatment, and management. Infect Drug Resist.

[4] Crotty KM, Kabir SA, Chang SS, Mehta AJ, Yeligar SM (2024). Pioglitazone reverses alcohol-induced alterations in alveolar macrophage mitochondrial phenotype. Alcohol Clin Exp Res (Hoboken).

[5] Yang X, Zeng Z, Liao Z (2024). Comparison of proximal gastrectomy and total gastrectomy in proximal gastric cancer: a meta-analysis of postoperative health condition using the PGSAS-45. BMC Cancer.

[6] Griffiths TL, Nassar M, Soubani AO (2020). Pulmonary manifestations of gastroesophageal reflux disease. Expert Rev Respir Med.

[7] Supriya BG, Harisree S, Savio J, Ramachandran P (2019). Actinomyces naeslundii causing pulmonary endobronchial Actinomycosis - A case report. Indian J Pathol Microbiol.

[8] Balis E, Kakavas S, Kompogiorgas S, Kotsifas K, Boulbasakos G (2019). Presentation of pulmonary tuberculosis and actinomyces co-infection as a lung mass: a literature review and unique case report. Monaldi Arch Chest Dis.

[9] Tanaka S, Araki H, Yoshizako T, Kitagaki H, Isobe T (2020). Pulmonary actinomycosis mimicking pulmonary cancer on fluorine-18 fluorodeoxyglucose PET-CT. Cureus.

[10] Asif AA, Roy M, Ahmad S (2021). More than valley fever: pulmonary actinomycosis and coccidioidomycosis co-infection in a patient. Eur J Case Rep Intern Med.

[11] Cliffe A, Hassan W, Ward D, Elgara M (2022). Actinomyces meyeri causing disseminated actinomycosis in the presence of concurrent bronchogenic carcinoma. BMJ Case Rep.

[12] Thapa K, Sarker M, Graman P (2022). Thoracoabdominal actinomycosis associated with laparoscopic cholecystectomy and mimicking metastatic pulmonary malignancy. BMJ Case Rep.

[13] Aydin Y, Arslan R, Filik M (2022). Pulmonary actinomycosis mimicks lung cancer. Rev Soc Bras Med Trop.

[14] Afsin E, Bacaksu E (2022). Endobronchial actinomycosis: a case report. Niger J Clin Pract.

[15] Ferreira M, Ferreira L, Amorim Pereira I, Santos Silva A, Henriques Ferreira I (2023). Pulmonary actinomycosis: a diagnostic challenge. Cureus.

[16] Rouis H, Moussa C, Ayadi A, Khattab A, Khouaja I, Zendah I, Maâlej S (2023). Pulmonary actinomycosis mimicking lung malignancy: About two cases. Heliyon.

[17] Cuzzani G, Fortunati E, Zanoni L (2024). Case report: pulmonary actinomyces infection mimics lung cancer on [(68)Ga]Ga-FAPI PET/CT. J Nucl Med.

[18] Bhat A, J M, Mascarenhas D (2024). Bronchiectatic actinomycosis with osseous metaplasia masquerading as lung cancer. Turk Patoloji Derg.

[19] Zheng M, Wei Y, Borkar NA, Hong X, Yang X, Zhang N, Wang X (2024). Pulmonary actinomycosis masquerading as lung cancer. Respir Med Case Rep.

[20] Wu S, Qin Z (2024). Pulmonary actinomycosis misdiagnosed as lung cancer: a case report. J Int Med Res.

